# Reduced FRG1 expression promotes angiogenesis via activation of the FGF2‐mediated ERK/AKT pathway

**DOI:** 10.1002/2211-5463.13582

**Published:** 2023-03-31

**Authors:** Bratati Mukherjee, Pratush Brahma, Talina Mohapatra, Saurabh Chawla, Manjusha Dixit

**Affiliations:** ^1^ Cancer and Angiogenesis Research Lab, School of Biological Sciences National Institute of Science Education and Research Bhubaneswar India; ^2^ Training School Complex Homi Bhabha National Institute Anushaktinagar India

**Keywords:** angiogenesis, breast cancer, ERK/AKT signaling, FGF2, FRG1, HUVECs

## Abstract

Identifying novel targets that control both tumorigenesis and angiogenesis can aid in developing a more potent anti‐angiogenic therapeutic strategy. We previously reported that reduction of FRG1 is associated with increased p38‐MAPK signaling in prostate cancer and with elevated MEK–ERK signaling in breast cancer. Here, we reveal the role of FRG1 in tumor angiogenesis. Our findings demonstrate that depleted FRG1 levels enhance the proliferation, migration, and tubule formation of HUVECs in a paracrine manner, and this was further substantiated in multiple animal models. Mechanistically, FRG1 depletion activated the expression of FGF2 in breast cancer cells, which triggered the ERK/AKT cascade in endothelial cells. As FRG1 affects multiple tumorigenic properties and it is upstream of FGF2, it can be explored as a therapeutic target that is less prone to resistance.

## Graphical abstract

The graphical representation shows that the depletion of FRG1 in breast cancer cells enhances the level of FGF2. This FGF2 binds to the FGF receptors, expressed on the HUVECs, which in turn activates the ERK‐ and AKT‐mediated downstream angiogenic cascade.

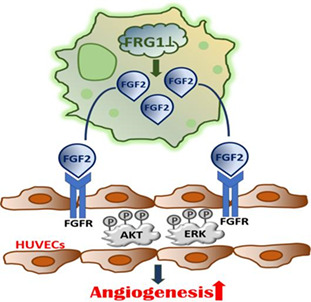

**Abbreviations**
CAM: chick chorioallantoic membrane assay
FGF2: basic fibroblast growth factors
FGFR: fibroblast growth factor receptor
FRG1: FSHD region gene 1
HUVECs: human umbilical vein endothelial cells
VEGF: vascular endothelial growth factors

Breast cancer is reported as the most common malignancy (24.5%) and the primary cause of cancer‐related deaths (15.5%) among women [1]. Progression of breast cancer is concurrent with increased neovascularization or angiogenesis. Breast cancer cells regulate angiogenesis by secreting various pro‐angiogenic factors such as vascular endothelial growth factors (VEGF), basic fibroblast growth factors (FGF2), interleukins, platelet‐derived growth factor (PDGF), transforming growth factors β (TGFβ), and tumor necrosis factors (TNF) [2, 3]. So far, anti‐angiogenic treatment of cancer largely depends on the inhibition of VEGF or its receptor, but numerous side effects and resistance have become a setback [4]. In long term, inhibition of VEGF receptors (VEGFR) with bevacizumab, sorafenib, and sunitinib results in drug resistance via activation of crucial oncogenic signaling such as MAPK, AKT, and EGFR [5]. Similarly, treating patients with the inhibitors of the FGF signaling cascade results in an inadequate clinical benefit due to the activation of other signaling pathways such as MEK/ERK and AKT [6, 7]. Although the combination of dual tyrosine kinase inhibitors that target both VEGF and FGF receptors has been proven to be more efficacious in delaying the resistance, crosstalk between the two signaling often fails to attenuate the process of angiogenesis [8]. Blockade of VEGFR2 initially exhibited a response in the pancreatic mouse model, but later started expressing elevated FGF2 levels [9]. Hence, further investigation into the underlying molecular mechanism of other potential angiogenic mediators that act upstream of these known signaling molecules is crucial in developing a better therapeutic strategy to evade the acquired resistance.

FSHD region gene 1 (FRG1), which was first identified as a candidate gene for facioscapulohumeral muscular dystrophy (FSHD), has been reported to have a role in muscle development [10]. In the recent past, its reduced level was reported in oral, gastric, colorectal, and prostate cancers [11, 12]. In prostate cancer, depleted FRG1 levels increased cell proliferation, migration, and invasion via activation of the p38‐MAPK pathway [12]. Moreover, FRG1 acts as a transcriptional repressor of GM‐CSF and suppresses the downstream ERK‐mediated EMT progression in breast cancer [13]. Interestingly, a few early studies indicated the possible role of FRG1 in angiogenesis either indirectly or in the Xenopus model [14]. The direct association of FRG1 in tumor angiogenesis was first established by Tiwari *et al*. [11], who showed that elevated expression of FRG1 in HEK 293T cells decreased tubule formation and migration of human umbilical vein endothelial cells (HUVECs) in a paracrine manner, but the mechanistic insights are unknown. This study was taken up to explore the exact role of FRG1 in tumor angiogenesis, and its mechanistic attribute to ascertain if it is upstream of VEGF A or FGF2.

To check whether the effect of FRG1 was similar regardless of breast cancer molecular subtypes, we perturbed the expression of FRG1 in breast cancer cell lines of different origins and used the conditioned media to study its effect on human endothelial cell properties relevant to angiogenesis. *In vitro* findings were further proven by *the ex‐ovo* Chick chorioallantoic membrane assay (CAM) assay, matrigel plug, and skin wound‐healing assay in mice. Mechanistically, we showed that FRG1 acts on upstream of FGF2, which eventually activated the AKT/ERK signaling axis in endothelial cells leading to angiogenesis induction. This work provides a better knowledge on understanding the role of FRG1 in cancer angiogenesis and may open up new therapeutic approaches which function irrespective of VEGF A/FGF2 signaling, reducing the possibility of resistance development in antiangiogenic therapy.

## Material and methods

### Cell culture, plasmid, and generation of stable cell line

Human Breast cancer cell lines MCF7 and MDA‐MB‐231 were procured from the cell repository, National Center for Cell Science (Pune, India). MCF7 cells were grown in Dulbecco's Modified Eagle Media (DMEM; Himedia, Mumbai, India) supplemented with 1X Penicillium‐Streptomycin‐Amphotericin B (PSA; Himedia) and 10% fetal bovine serum (FBS; Himedia, US origin). MDA‐MB‐231 cells were grown in Roswell Park Memorial Institute Media (RPMI; Himedia) supplemented with 1× PSA (Himedia) and 15% FBS (Himedia, US origin). HUVECs were purchased from Lonza (Walkersville, MD, USA) and maintained in complete Endothelial Cell Growth Media‐2 (EGM‐2, Lonza, Walkersville, MD, USA). Cells were grown in the incubator (Eppendorf, Hamburg, Germany) at 37 °C temperature and 5% CO_2_.

### Generation of stable cell lines

FRG1 knockdown (pLKO.1_FRG1sh, TRCN0000075012) and expression vectors (HsCD004 21091, PLX304_FRG1) were purchased from Sigma‐Aldrich (St. Louis, MO, USA) and Harvard Repository (Cambridge, MA, USA), respectively. Cells were transfected using Lipofectamine 3000 (Invitrogen, Van Allen Way Carlsbad, CA, USA) as per the manufacturer's protocol and selected on puromycin (2 μg·mL^−1^; Sigma Aldrich, St. Louis, MO, USA) and blasticidin (10 μg·mL^−1^; Sigma Aldrich, St. Louis, MO, USA) for knockdown and expression vectors along with their control vectors, respectively. The generation of stable single cell derived lines was confirmed by Western blotting.

### Preparation of conditioned media

MCF7 and MDA‐MB‐231 cells (2 × 10^6^) with perturbed FRG1 levels, and their respective controls, were cultured in a 100 mm dish in DMEM and RPMI, respectively, with 2% FBS. After 96 h, conditioned media (CM) was collected in a 15‐mL tube and centrifuged at 1792 *g* for 5 min at 4 °C. The supernatant was collected in a fresh tube, aliquoted, and stored at −80 °C till further use.

### Tubule formation assay

Matrigel tubule formation assay was performed in μ‐slide angiogenesis plate (Ibidi, Munich, Germany). Growth factor reduced matrigel (Corning, NY, USA) (10 μL per well) was added to each well of the slide and allowed to solidify by placing the plate in a humidified chamber at 37 °C for an hour. The cell suspension was prepared using 7000 HUVECs in EGM‐2 (Lonza, Walkersville, MD, USA) and conditioned media in a 1 : 1 ratio following the previously established protocol [15]. After 6 h, images were taken at ×10 magnification in an inverted microscope (Nikon, Tokyo, Japan). Images were analyzed with the Angiogenesis Analyzer plugin of ImageJ software (NIH, Bethesda, MD, USA).

### MTS assay for cell proliferation

HUVECs were seeded (5000 cells per well) in a 96‐well plate into 200 μL of complete EGM‐2 (Lonza, Walkersville, MD, USA). Post 24 h of seeding, old media was replaced with a cocktail of EGM‐2 and conditioned media (1 : 1 ratio) [15]. After 24 h, the cocktail was replaced with 100 μL of fresh EGM‐2 media and 10 μL of CellTiter 96® AQueous One Solution Reagent (Promega, Madison, WI, USA) and incubated for an hour inside the incubator at 37 °C and 5% CO_2_. Afterward, absorbance was recorded in Varioscan multimode microplate reader (Thermo Fisher Scientific, Waltham, MA, USA) at 490 nm.

### Transwell migration assay

For the Transwell migration assay, 0.5 × 10^6^ HUVECs were suspended into a cocktail of 0.5 mL of conditioned media (harvested from FRG1 depleted MCF7 cells and MDA‐MB‐231 cells with ectopic expression of FRG1) and 0.5 mL of EGM‐2 growth media, and plated onto the membrane filter inserts of 8 μm pore size (Merck, Billerica, MA, USA). Inserts were kept in a 12‐well plate where the lower chambers were filled with 1 mL of EGM‐2 growth media and kept at 37 °C for 24 h in a humidified chamber containing 5% CO_2_. After 24 h, inserts were taken out, and cells were fixed with methanol (Merck, Mumbai, India), followed by staining with Giemsa (Himedia). Nonmigrated cells were gently removed with a cotton bud. Images were taken at ×10 magnification in an upright brightfield microscope (Olympus, Tokyo, Japan).

### Western blot

Cells were washed with PBS, and the lysate was prepared using ice‐cold RIPA buffer (Thermo Scientific, Rockford, IL, USA), supplemented with protease‐phosphatase inhibitor (Thermo Scientific, Rockford, IL, USA). Protein quantification was carried out using BCA reagent (Thermo Scientific, Rockford, IL, USA) according to the manufacturer's protocol. Protein samples were prepared in a 4× Laemmli buffer and boiled at 100 °C for 5 min. Around 20–30 μg of protein was separated on 12% SDS/PAGE and transferred onto a poly‐vinylidene fluoride (PVDF; Millipore, Bangalore, India) membrane and probed with primary antibodies (Table S1) overnight, followed by 1‐h incubation with respective horseradish peroxidase (HRP)‐conjugated secondary antibodies (Abgenex, Bhubaneswar, India). Subsequently, a chemiluminescence signal was developed using SuperSignal™ West Femto maximum sensitivity substrate (Thermo Scientific, Rockford, IL, USA), and bands were detected in Chemidoc XRS+ (Bio‐Rad, Hercules, CA, USA). ImageJ (NIH, Bethesda, MD, USA) software was used to analyze the images.

### RNA extraction and quantitative real time PCR

Total RNA was isolated from the cells using RNeasy mini kit (Qiagen, Hilden, Germany) following the manufacturer's protocol. cDNA was prepared with one μg of RNA using the verso cDNA synthesis kit (Thermo Scientific, Vilnius, Lithuania, Europe). For each experimental condition, qPCR reaction was performed in triplicate using 10 ng of cDNA, 2x SYBR Green PCR Master Mix (Applied Biosystem, Austin, TX, USA) and respective primers (Table S2) in Applied Biosystem 7500 system (ThermoFisher, Waltham, MA, USA). GAPDH was used as the internal control. The ΔΔ*C*_t_ method was used to calculate the relative expression of the transcript.

### Enzyme‐linked immunosorbent assay (ELISA)

The quantity of VEGF A, present in the supernatant of MCF7 cells with depleted FRG1 and MDA‐MB‐231 cells with ectopic FRG1 expression, was measured using the Human VEGF Quantikine ELISA Kit (R&D Systems, MN, USA). Briefly, 1 × 10^6^ cells were plated into a 100 mm dish in complete cell culture media. On the next day, the entire media was replaced by the serum‐free media and incubated for the next 24 h. Subsequently, the supernatant was collected and centrifuged at 1792 *g* for 10 min at 4 °C to eliminate the debris. This supernatant was used to carry out the ELISA as per the manufacture's (R&D Systems, Minneapolis, MN, USA) instruction. OD value was taken at 450 nm in the Varioscan multimode microplate reader (Thermo, Waltham, MA, USA).

### Matrigel plug assay

The experiment was approved by the Institutional Animal Ethics Committee, NISER (Protocol No. NISER/SBS/IAEC/AH 109). All the animals used during this study were housed in autoclaved polysulfone cages with corncob bedding in a controlled environment with temperature and humidity ranging between 22 ± 3 °C and 40–70%, respectively. Animals were exposed to artificial lighting with a 12‐h light/12‐h dark cycle as a routine practice. Purified UV‐treated drinking water was provided to the animals. Animals were fed with a standard commercially available pellet diet. Water and feed were provided ad‐libitum. The experiments were planned in accordance with the 3Rs principles of reduction, replacement, and refinement for animal studies. All the *in vivo* experiments were carried out under the supervision of a trained veterinarian. Optimal standardized surgical procedures were carried out on the test animals under the surgical plane of anesthesia, thereby reducing the stress and suffering. Animals were monitored routinely by the veterinarian and animal care staff for signs of pain or distress. A cocktail of condition media and matrigel (1 : 1 ratio) was injected subcutaneously into the right flank of 6–7‐week‐old female C57BL/6 mice. After 7 days, mice were euthanized in the CO_2_ gas chamber, and matrigel plugs were excised out. The plugs were photographed in a digital camera and fixed in formalin.

### Chick chorioallantoic membrane assay (CAM)

To perform the CAM, 3‐day‐old fertilized eggs were purchased from Central Poultry Development Organization (Bhubaneswar, India). The outer surface of the eggs were thoroughly cleaned in sterile water and 70% ethanol. Eggs were then kept inside an incubator at 37 °C and 50% humidity for a day. Eggs were broken using a metal forcep, and the intact embryo was placed in transparent plastic cups, covered with the transparent cling wrap, as described by Naik *et al*. [16]. A circular filter paper disc (1 mm thick and 0.04 mm in diameter) was placed over the CAM using a sterile forcep. After 4 days, CM was applied to the paper disc. The cups were covered with cling wrap and placed inside the incubator. After 7 days of incubation, images of the blood vessels around the filter disc were captured in a digital camera. Number of microvessels that arise from the centre of the filter disc were counted manually.

### Skin wound‐healing assay in mice

Skin wound‐healing assay in mice was performed after taking approval from the institutional animal ethics committee, NISER (NISER/SBS/IAEC/AH 109). Six‐ to eight‐week‐old female BALB/c mice, devoid of any kind of skin infection or injury, were only selected for the experiments. A wound between 4 and 6 mm in diameter was made with a punching machine, and a silicone splint with an inner diameter of 8 mm and a thickness of 0.5 mm was placed around the wound with adhesive and 3–0 nonabsorbable sterile surgical suture (Johnson and Johnson PVT. LTD, Maharastra, India). A dressing film (3M Tegaderm India Limited, Maharastra, India) was used to conceal the wound. The mice were administrated with the analgesic Tramadol (50 mg·kg^−1^ body weight) intraperitoneally every 12 h for 2 days. Mice of each group were applied 100 μL of respective conditioned media and 100 μL of growth factor reduced matrigel (1 mg·mL^−1^; Corning) in the middle of the wound with an interval of 12 h for 9 days, starting from the day of wound creation. Images of each animal and the wounds were captured using a digital camera on every third day till the day of sacrifice (on the ninth day). ImageJ software was used to measure the percentage of wound closure. The rate of wound healing was calculated using the formula: (Wound area on day 0‐Wound area on a respective day) × 100/Wound area on day 0. Details of the animal maintenance have been discussed in the ‘Matrigel plug assay’ section.

### Inhibition of FGF receptor (FGFR) by pharmacological compound

HUVECs (1 × 10^6^) were seeded in a six‐well plate and treated with the cocktail of EGM2 and CM in a 1 : 1 ratio. After 24‐h incubation, cells were treated with 100 nm of FGFR inhibitor (Infigratinib MedChemExpress, Monmouth Junction, NJ, USA) for 6 h (dose and time optimized), and the lysates were harvested to perform the downstream experiments.

### Statistical analysis

Statistical analysis was performed using graphpad prism  6.0 version (GraphPad Software Inc., Boston, MA, USA) and Microsoft Excel (Microsoft, Redmond, WA, USA). Two‐tailed, unpaired Student's *t*‐test was used to calculate the statistical difference between the mean of two groups. *P*‐value ≤0.05, was considered to be significant for all the tests.

## Result

### Depleted FRG1 levels in breast cancer cells promoted the proliferation and migration of HUVECs

We generated stable cell lines with FRG1 level modulation in MCF7 (estrogen receptor positive cells; ER+) cells that harbors moderate expression of endogenous FRG1 and MDA‐MB‐231 (Triple negative breast cancer cells; TNBC) cells that has the basal level of endogenous FRG1. As MCF7 has more endogenous FRG1 than MDA‐MB‐231 cells, we established the stable MCF7 cells with depleted FRG1 level (FRG1_KD) and MDA‐MB‐231 cells with ectopic expression of FRG1 (FRG1_Ex).

**Fig. 1:**
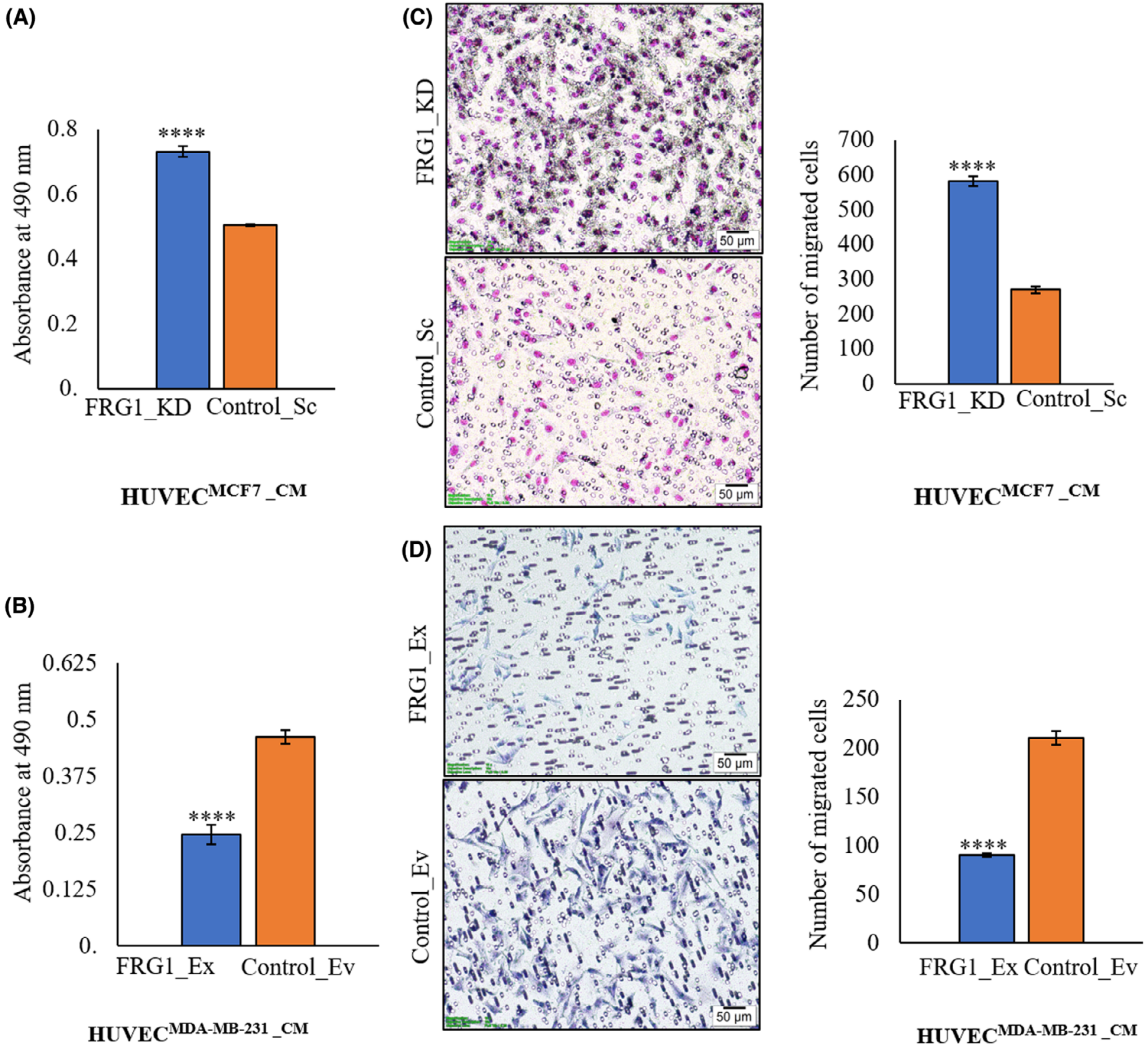
Depletion of FRG1 in breast cancer cells promotes HUVECs proliferation and migration. The expression of FRG1 was altered in MCF7, and MDA‐MB‐231 cells and the conditioned media (CM) was harvested. HUVECs were treated with the CM and subjected to proliferation and migration assays. (A, B) Bar diagrams show MTS assay based OD values taken at 490 nm at 24 h for HUVECs incubated in the CM obtained from (A) MCF7 cells with FRG1 knockdown (FRG1_KD) and corresponding control (Control_Sc), and from (B) MDA‐MB‐231 cells with ectopic expression of FRG1 (FRG1_Ex) and corresponding control (Control_Ev). (C) Representative images of transwell migration assay of HUVECs grown in the CM from MCF7 cells with FRG1 depletion (FRG1_KD) and corresponding control (Control_Sc). Bar diagram depicts the no. of migrated cells at 24 h. Images were taken at ×10 magnification. Scale bar: 50 μm. (D) Representative images of transwell migration assay of HUVECs grown in CM, obtained from MDA‐MB‐231 cells with ectopic expression of FRG1 (FRG1_Ex) and corresponding control (Control_Ev). The bar diagram shows the number of migrated HUVECs in the two groups. Images were taken at ×10 magnification. Scale bar: 50 μm. Experiments were performed in triplicate. Two‐tailed unpaired Student's *t*‐test was used to compare the significance of the differences between the groups. Results are presented as mean ± SD. ****P ≤ 0.0001.

MTS and transwell migration assays were performed to elucidate the effect of altered FRG1 expression in breast cancer cells on endothelial cell proliferation and migration. HUVECs were cultured in the conditioned media isolated from MCF7 and MDA‐MB‐231 with altered FRG1 expression. Conditioned media from MCF7 cells with depleted FRG1 expression induced HUVECs proliferation (Fig. 1A). An opposite trend was observed due to ectopic expression of FRG1 in TNBC cell line MDA‐MB‐231 (Fig. 1B). Similarly, the transwell migration assay showed increased migration of HUVECs when grown in the conditioned media from MCF7 with reduced FRG1 level (Fig. 1C). Parallelly, we observed decreased migration of HUVECs cultured in the conditioned media from MDA‐MB‐231 with elevated FRG1 expression (Fig. 1D).

Together, these data suggest that altered FRG1 levels can modulate the proliferation and migration of HUVECs, two crucial properties of angiogenesis.

### Paracrine effect of reduced FRG1 expression in breast cancer cells on tubule formation in HUVECs

**Fig. 2:**
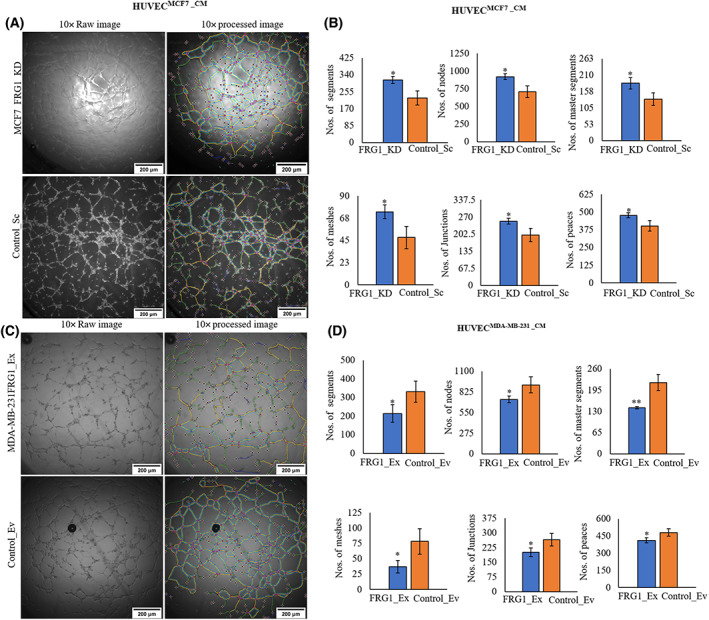
Reduced FRG1 level increases tubule formation ability of HUVECs. HUVECs were grown in the conditioned media (CM), harvested from MCF7 cells and MDA‐MB‐231 cells with altered FRG1 levels. After 6 h of incubation, images of the tubules were captured at ×4 magnification and further analyzed by the Angiogenesis analyzer tool of the ImageJ software. (A) Representative raw (left panel) and processed skeletonized images (right panel) showing tubule forming ability of HUVECs, treated with the CM from MCF7 cells with reduced FRG1 (FRG1_KD), and corresponding control (Control_Sc). (B) Bar diagram showing the difference of various tubule forming parameters between MCF7_FRG1_KD and Control_Sc. (C) Representative raw (left panel) and processed skeletonized images (right panel) showing tubule forming ability of HUVECs, treated with the CM from MDA‐MB‐231 cells with elevated expression of FRG1 (FRG1_Ex), and corresponding control (Control_Ev). (D) Bar graphs showing the difference in various tubulogenic parameters between the two groups. Scale bar: 100 μm. Experiments were performed in triplicate. Two‐tailed unpaired Student's *t*‐test was used to compare significance of the differences between groups. Results are presented as mean ± SD. *P ≤ 0.05; **P ≤ 0.01.

To identify the effect of FRG1 modulation on endothelial cell differentiation, tubule formation assay was done. We used a co‐culture setup of HUVECs and conditioned media harvested from MCF7 and MDA‐MB‐231 cells with perturbed FRG1 levels. Treatment of HUVECs with conditioned media obtained from FRG1 depleted MCF7 cells led to increased tubule formation in HUVECs (Fig. 2A). This observation was further evident by quantitative analysis that revealed number of segments, number of nodes, number of master segments, number of meshes, number of junctions, and number of peaces were increased significantly (Fig. 2B). Other angiogenesis properties including number of master junctions, total master segment length, total meshes area, total branching length, total segment length, and branching interval showed trend towards increased levels (Fig. S1). In contrast, HUVECs grown in conditioned media obtained from MDA‐MB‐231 with elevated FRG1 expression showed reduced tubule forming ability (Fig. 2C). Significant decrease was observed in the number of segments, number of nodes, number of master segments, number of meshes, number of junctions, and number of peaces (Fig. 2D). We also found a trend towards reduced levels of number of master junctions, total master segment length, total meshes area, total branching length, total length, total segment length, and branching interval in HUVECs treated with the conditioned media from MDA‐MB‐231 with increased FRG1 expression (Fig. S2). Overall, these results show pro‐angiogenic potential of tumor cells with reduced expression of FRG1.

### Low FRG1 level correlated with increased angiogenesis in animal model

**Fig. 3:**
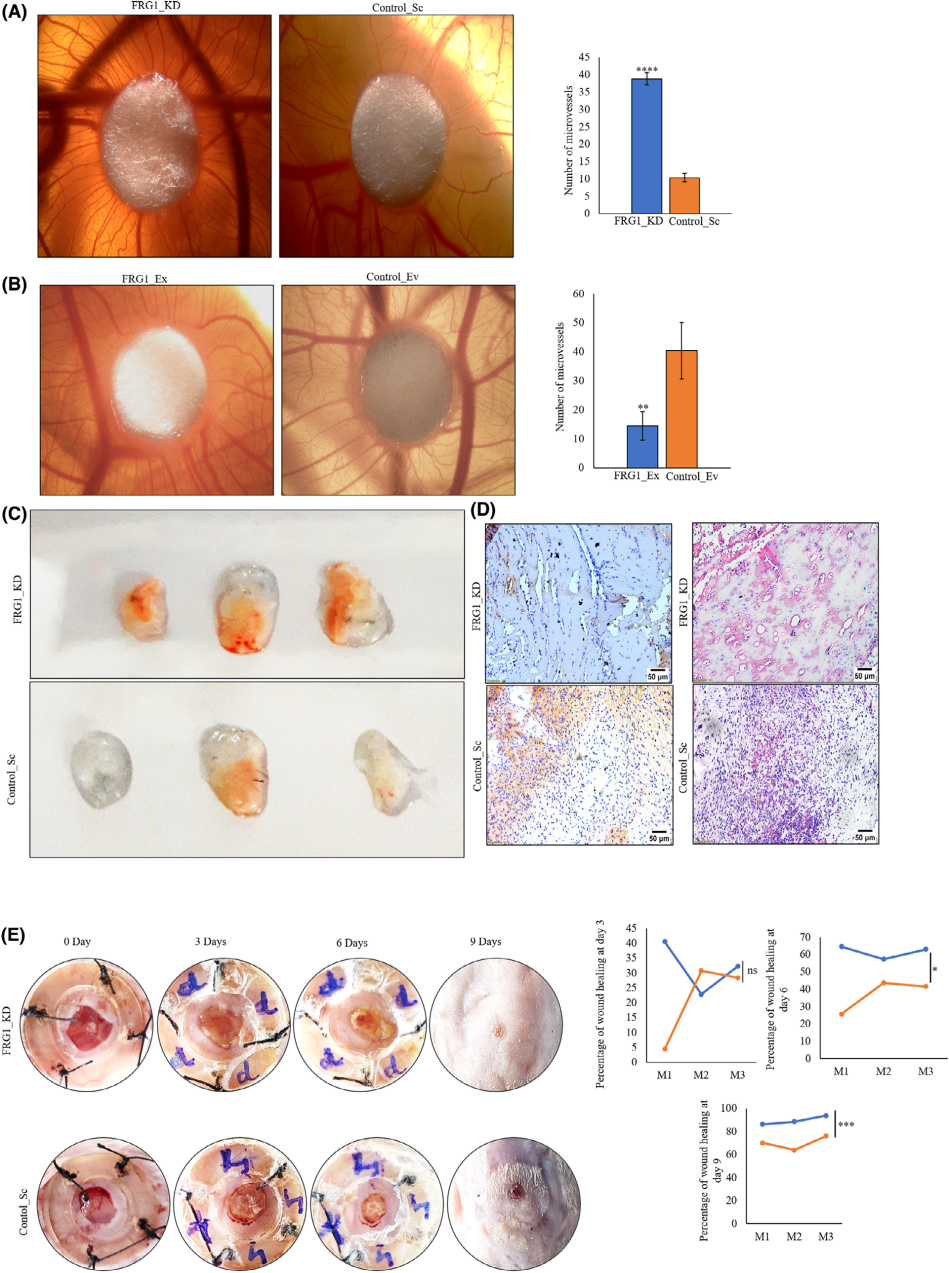
FRG1 expression in breast cancer cells affects angiogenesis *in vivo*. Conditioned media (CM) was harvested from MCF7 or 4T1 cells with altered FRG1 expression, and used for various animal models based experiments to check the angiogenic potential of FRG1. (A) Representative CAM images showing the microvessels around the paper disc soaked in the CM from FRG1 depleted MCF7 cells (FRG1_KD) along with the control (Control_Sc). The images were taken on day 9^th^ using a stereo microscope. Bar diagram showing the no. of microvessels in the two groups. *n* = 7. (B) Representative CAM images, indicating the microvessels around the paper disc soaked in CM from FRG1 expressing MCF7 cells (FRG1_Ex) and its control (Control_EV). The bar diagram depicts the no. of microvessels in the two groups. *n* = 4. (C) Representative images depict the gross overview of matrigel plugs (excised on 7^th^ day from C57 BL/6 mice) that were treated with matrigel and CM from FRG1 depleted MCF7 cells (FRG1_KD) and control (Control_Sc). *n* = 3. (D) The left panel shows the representative IHC images of the microvessels of the matrigel plugs stained with CD31 antibody, treated with the CM harvested from FRG1 depleted MCF7 cells (FRG1_KD) and Control_Sc. The right panel shows the H&E images of the same set. Images were taken at ×4 magnification. Scale bar 50 μm. (E) Representative images showing the wound‐healing process in BALB/c mice treated with CM from 4T1 cells with FRG1 depletion (FRG1_KD) and corresponding control (Control_Sc) on different days. Graph showing the percentage of wound recovery between the mice treated with FRG1 depleted (FRG1_KD) 4T1 cells and the control (Control_Sc) group. *n* = 3. Two‐tailed unpaired student's *t*‐test was used to compare the significance of difference between the two groups. ^ns^ *P* > 0.05, *P ≤ 0.05; **P ≤ 0.01, ***P ≤ 0.001, ****P ≤ 0.0001.

To demonstrate the effect of FRG1 expression on tumor angiogenesis *in vivo*, we validated our *in vitro* findings in multiple animal models. Conditioned media from MCF7 with reduced FRG1 expression induced more microvessels from CAM (Fig. 3A). Parallel to our cell‐based observation, we found a lesser number of microvessels in the CAM treated with the conditioned media from MCF7 cells with an elevated level of FRG1 than the control (Fig. 3B).

Furthermore, matrigel plug assay in C57/BL6 mice showed increased vascularity of plugs treated with the conditioned media from MCF7 with depleted FRG1 levels (Fig. 3C). Immunohistochemistry with CD31 antibodies and H&E staining of the plugs also confirmed a significantly increased microvessels count (Fig. 3D). The above findings support that reduction of FRG1 may lead to increased angiogenesis that was further confirmed by skin wound‐healing assay in BALB/c mice. Wounds treated with the conditioned media harvested from 4T1 cells with depleted FRG1 expression healed more quickly than wounds in the control group (Fig. 3E). By the ninth day, there was significantly more wound recovery.

Collectively, this findings indicate the potential of FRG1 depletion to induce angiogenesis *in vivo*.

### FRG1 modulation in breast cancer cells altered the expression of FGF2

**Fig. 4:**
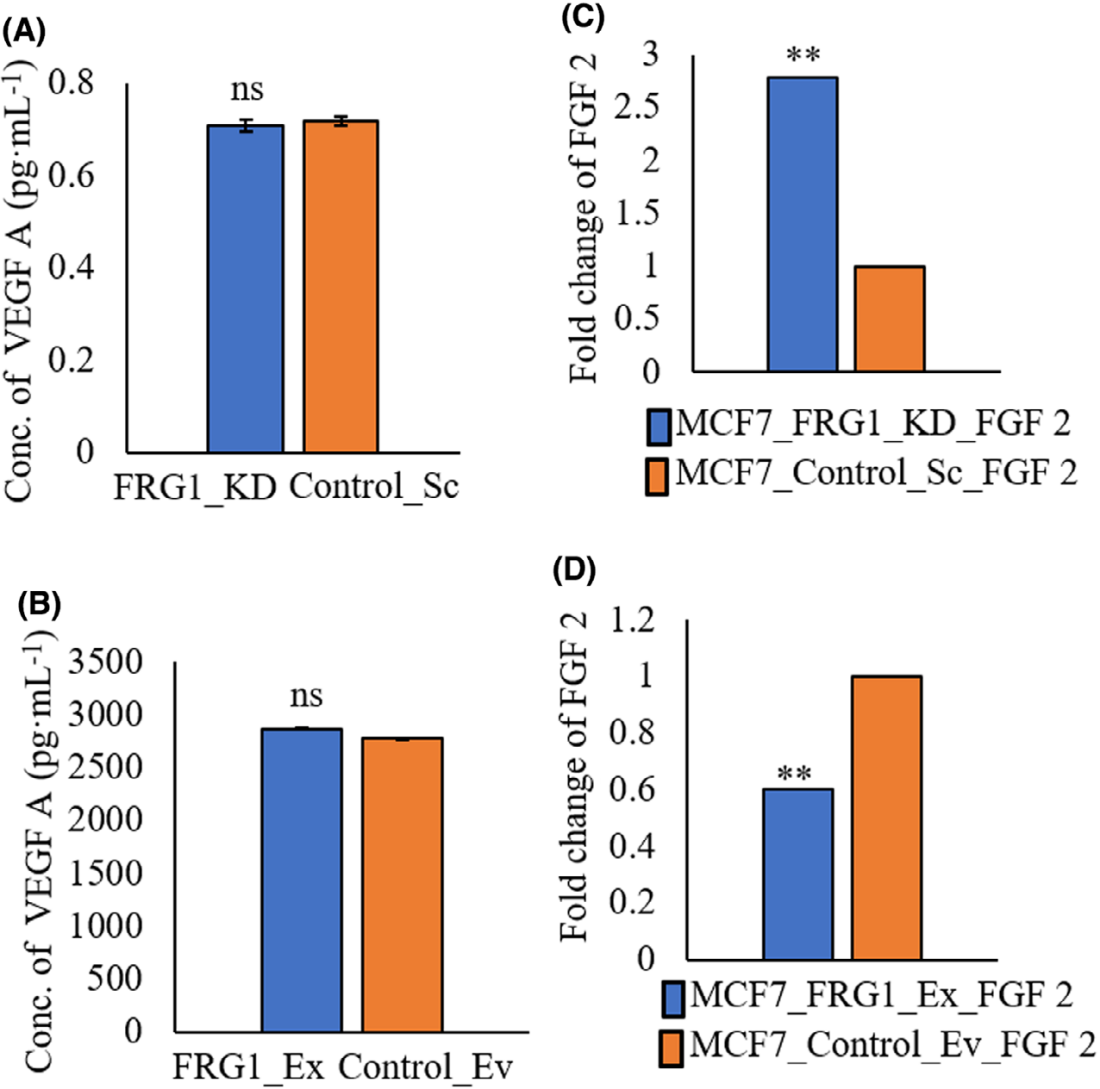
FRG1 depletion increases the expression of angiogenic cytokines. (A) Representative bar graph of ELISA showing the effect of FRG1 reduction in MCF7 (FRG1_KD vs. Control_Sc) on the levels of VEGF A in the conditioned media. OD was taken at 490 nm. (B) Bar graphs of ELISA showing the effect of ectopic expression of FRG1 in MDA‐MB‐231 cells (FRG1_Ex) on the protein level of VEGF A as compared to control (Control_Ev). (C) Bar graphs showing the difference in transcript level of FGF2, due to reduced expression of FRG1 in MCF7 cells (FRG1_KD) compared to control (Control_Sc). (D) Bar diagrams depict the difference in relative mRNA level of FGF2 due to increased FRG1 expression in MCF7 cells (FRG1_Ex) compared to its control (Control_Ev). Experiments were performed in triplicate. Two‐tailed unpaired Student's *t*‐test was used to compare the significance of difference between the two groups. Results are presented as mean ± SD. ^ns^ *P* > 0.05, **P ≤ 0.01.

VEGF A and FGF2 are the two most potent regulators of angiogenesis [17]. To get insights into the molecular mechanism behind FRG1‐mediated tumor angiogenesis, we first examined the effect of FRG1 perturbation on VEGF A by ELISA. We did not observe any changes in VEGF A levels in the conditioned media of MCF7 and MDA‐MB‐231 cells with perturbed FRG1 expression (Fig. 4A,B), but FGF2 mRNA expression was affected. Depletion of FRG1 level in MCF7 led to increased FGF2 expression (Fig. 4C). Similarly, ectopic expression of FRG1 in MCF7 led to reduced levels of FGF2 transcripts (Fig. 4D).

Together, these data show that loss of FRG1 may promote angiogenesis via upregulating FGF2.

### Reduced FRG1 expression in breast cancer cells induced ERK‐AKT signaling in HUVECs

**Fig. 5:**
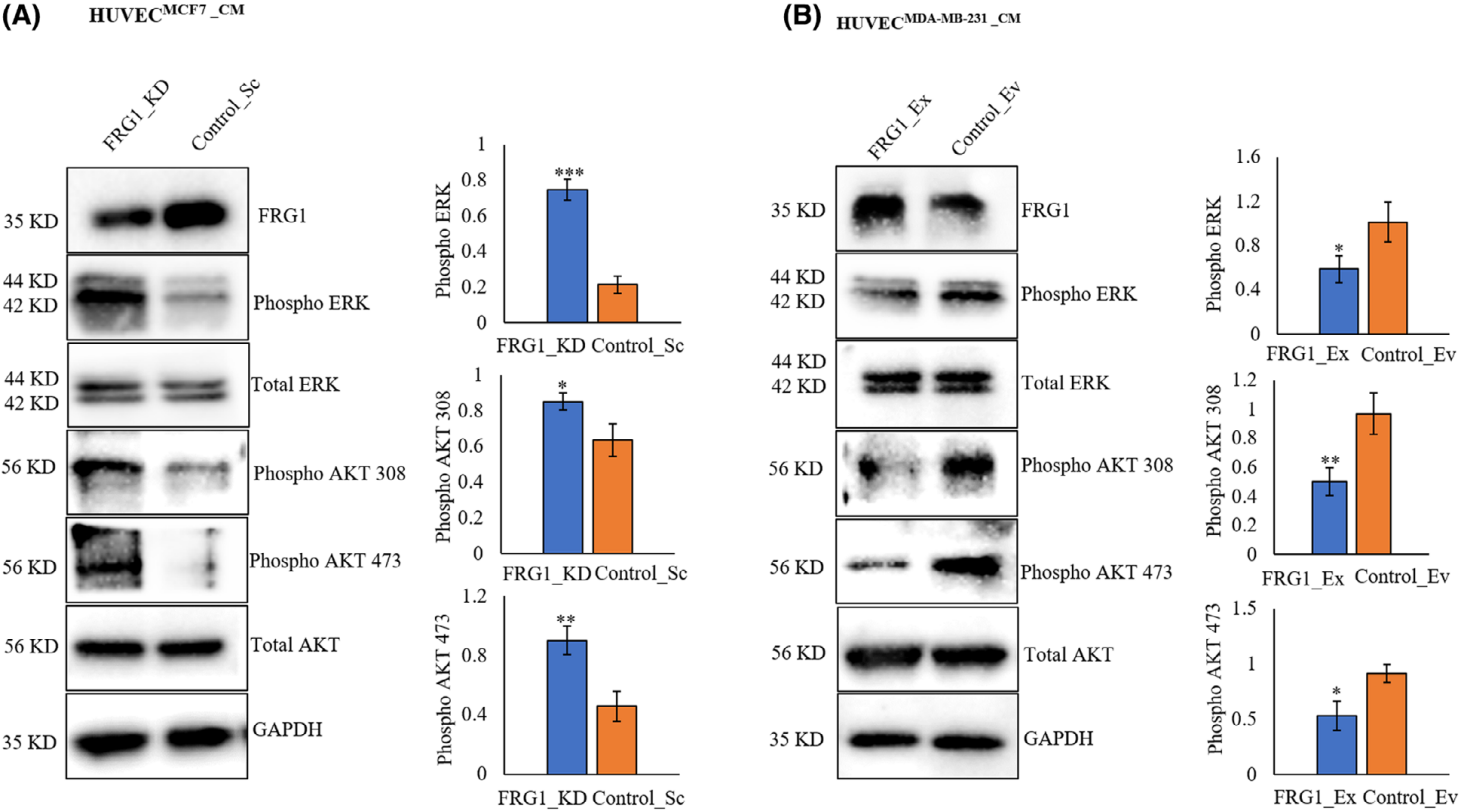
FRG1 depletion in breast cancer cells activates ERK and AKT in HUVECs. Conditioned media (CM) was collected from MCF7 and MDA‐MB‐231 cells with perturbed FRG1 expression, and HUVECs were incubated into it for 24 h. (A) Representative Western blot images showing activation of phospho‐ERK, phospho‐AKT 473/308 in HUVECs grown in the CM obtained from MCF7 cells with reduced expression of FRG1 (FRG1_KD) and control (Control_Sc). Bar diagrams depict the phospho‐ERK and phospho‐AKT 473/308 levels in the two groups. (B) Representative Western blot images showing phospho‐ERK, phospho‐AKT 473/308 in HUVECs grown in the CM obtained from MDA‐MB‐231 cells with ectopic expression of FRG1 (FRG1_Ex), compared to control (Control_Ev). Bar diagrams depict the phospho‐ERK and phospho‐AKT 473/308 levels in the two groups. GAPDH was used as the loading control. Experiments were performed in triplicate. Two‐tailed unpaired Student's *t*‐test was used to compare the two groups' significance of differences. Results are presented as mean ± SD. ^ns^ *P* > 0.05, *P ≤ 0.05; **P ≤ 0.01, ***P ≤ 0.001.

FGF2 is well known to activate the ERK and AKT signaling in endothelial cells [18, 19, 20]. To confirm the same, we treated HUVECs with the conditioned media from MCF7 with reduced FRG1 expression. Immunoblotting revealed an increased level of phospho‐ERK, phospho‐AKT 308/473 in HUVECs (Fig. 5A). As further confirmation of our study, we observed a significant downregulation in the activation of ERK and AKT 308/473, when HUVECs were grown in the conditioned media from MDA‐MB‐231with elevated expression of FRG1 (Fig. 5B). Overall, these data suggest that reduced FRG1 levels might be inducing breast cancer angiogenesis by the activation of the AKT/ERK signaling pathway in HUVECs.

### FGF receptor (FGFR) inhibition prevents the angiogenic effect of FRG1 depletion in breast cancer cells on HUVECs

**Fig. 6:**
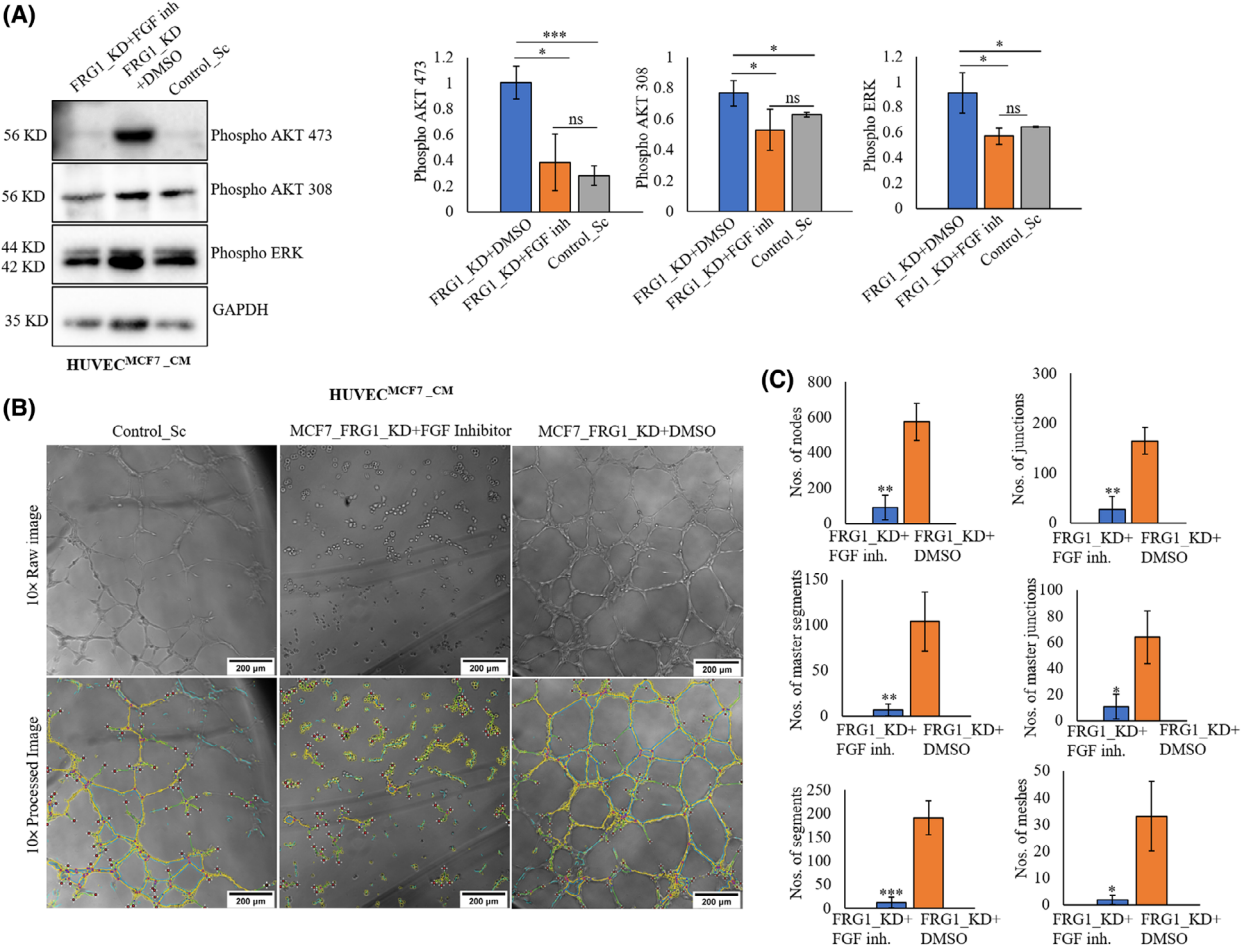
FGFR inhibition reduces FRG1 mediated activation of ERK and AKT. Conditioned media (CM) was collected from MCF7 cells with depleted FRG1 expression (MCF7_FRG1_KD) and the corresponding control (Control_Sc), and used for growing HUVECs along with FGF receptor (FGFR) inhibitor Infigratinib (100 nm) or DMSO control for 6 h. Thereafter protein lysate was harvested from HUVECs, and Western blot analysis was performed. GAPDH was used as the loading control. (A) Representative Western blot images and corresponding bar graphs show phospho‐ERK and phospho‐AKT 473/308 levels in HUVECs treated with MCF7_FRG1_KD + DMSO, MCF7_FRG1_KD + FGFR inhibitor, and MCF7_Control_Sc. (B) Images show tubule forming ability of HUVECs treated with MCF7_FRG1_KD + DMSO, MCF7_FRG1_KD + FGFR inhibitor, and MCF7_Control_Sc. (C) Bar graphs showing various tubulogenic parameters in the two groups. Experiments were performed in triplicate, two‐tailed unpaired Student's *t*‐test was used to compare the significance of the groups' differences. Results are presented as mean ± SD. ^ns^ *P* > 0.05, *P ≤ 0.05; **P ≤ 0.01; ***P ≤ 0.001. Scale bars: 200 μm.

As reduced FRG1 level enhanced the activation of ERK and AKT, next we checked whether it is mediated by FGF signaling. HUVECs cultured in conditioned media from FRG1 depleted MCF7 cells were simultaneously treated with FGFR inhibitor (Infigratinib). We observed that the abrogation of FGF2‐mediated ERK and AKT activation in HUVECs in the presence of FGFR inhibitor (Fig. 6A). Tubule formation assay also revealed a similar effect (Fig. 6B). Inactivation of the FGF pathway in HUVECs, grown in the conditioned media obtained from MCF7 cells with FRG1 knockdown, showed lesser tubules than the HUVECs treated with FGFR inhibitor solvent (Fig. 6B). We found a significant downregulation in the number of segments, number of nodes, number of junction, number of master segments, number of master junction, and number of meshes due to depletion of FRG1 (Fig. 6C). Other tubulogenic parameters such as total master segments, number of meshes, total meshes area, total length, total branching length, total segment length, branching interval, total branching length, total length, number of peaces, mesh index, and mean mesh size were also found to be reduced due to FGFR inhibitor treatment (Fig. S3).

These data suggest that reduced FRG1 level activates FGF that further increases the downstream phospho‐ERK and phospho‐AKT signaling in HUVECs.

## Discussion

Tumor growth and metastasis largely depend on angiogenesis that is triggered by various cytokines and growth factors secreted from tumor cells [21]. Lack of vascular support leads to tumor cell necrosis or even apoptosis [22, 23]. Among the various pro‐angiogenic growth factors and cytokines, members of FGF and VEGF superfamily are reported to be the most potent angiogenesis inducers [2]. In breast cancer, other cytokines such as IL‐6, IL‐1α, IL‐1β, IL‐8, TNF‐α/β, TGF‐β, and GM‐CSF are also known to stimulate angiogenesis [24, 25]. Although VEGF‐targeted chemotherapeutic drugs show significant results in patients, they often do not respond after a certain time either by evasive resistance in patients or by intrinsic resistance to VEGF blockers [26]. Therefore, targeting other angiogenic inducers that promote a strong angiogenic response such as FGF superfamily has emerged to be a focus of interest [27]. There are compelling evidences to support the role of FGF signaling in tumor angiogenesis [9, 28]. Multiple drugs have been approved by the FDA that target FGF or FGFR [29, 30]. But FGF inhibition therapy often activates other membrane signaling cascades EGFR, ERBB3, or MET that, in turn, fails to contribute to better patient survival [30]. Hence, finding the other upstream angiogenic regulators that can also control tumorigenic activities can aid in the comprehensive targeting of cancer.

The connection between FRG1 expression and angiogenesis originated from the finding observed in FSHD patients where 75% of FSHD patients show abnormalities in their retinal vasculature [31]. In spite of having a role as metastatic suppressor, involvement of FRG1 in tumor angiogenesis was mostly overlooked. In 2017, study by Tiwari et al. was the first direct indication of the possible role of FRG1 in human tumor angiogenesis [11]. Present work is a step ahead for establishing FRG1 as an angiogenic regulator that can be further explored to identify its therapeutic potential. We have found reduced FRG1 level leads to higher tubule formation and vice versa.

Mechanistically, we explored the effect of the two most potent angiogenic regulators, VEGF A and FGF2. Earlier, we reported no changes in VEGF A and FGF2 transcriptome level due to FRG1 level perturbation in HEK 293T cells [11]. Although our current data suggested no change in the level of VEGF A protein in FRG1 depleted MCF7 cells, the mRNA expression of FGF2 was altered. FGF signaling facilitates survival, proliferation, migration, and differentiation of endothelial cells via activating various signaling pathways through mainly four types of receptor tyrosine kinases, FGFR1, 2, 3, 4. Among the various isoforms of the FGF superfamily, FGF2 is the most potent angiogenic regulator [32]. Pro‐angiogenic effect of FGF2 has been established in various experimental models such as CAM, rabbit/mouse cornea, and matrigel plug assay [33]. Our findings indicate that the activation of FGF2‐mediated FGF signaling, brought on by the pro‐angiogenic factors present on the CM of FRG1 reduced cells alone, may be sufficient to cause HUVECs to exhibit elevated angiogenic characteristics. This observation is further supported by a previous study where FGF2 was found to be twice as potent as VEGF, in invading a collagen gel matrix and forming capillary‐like structures [34].

The process of angiogenesis requires coordinated molecular signaling facilitated mainly by the ERK‐AKT pathway. Activation of ERK signaling is needed in normal vascular development [35]. AKT signaling is pertinent in endothelial cell survival [36]. Migration of endothelial cells and formation of capillary‐like structures are largely dependent on the PI3K‐AKT pathway [37]. We found that FGF2 activates AKT and ERK both, in HUVECs, which is in parallel with previous studies [32]. FGF2 binds to the cell surface receptor heparan sulfate proteoglycans along with FGFR leading to activation of downstream signaling cascade Ras, Raf, MAPK, and ERK [38].

Planning therapeutic strategy to control FRG1 levels can be crucial in evading resistance mechanisms due to several reasons. First, FRG1 is known to affect multiple pathways, covering PI3K‐AKT‐P38 and ERK. Second, it is an upstream regulator of FGF2. Third, it has been reported to regulate multiple tumorigenic properties, including cancer cell proliferation, and EMT. Combination therapies including angiogenic inhibitors are already in use [39]. Single agent‐based anti‐angiogenic therapies are useful only in a subset of patients. Molecules with dual function may widen the coverage and improve the survivability.

In conclusion, the reduction in FRG1 increases FGF2 expression in breast cancer cells, which activates angiogenic properties of endothelial cells via AKT‐ERK signaling.

## Conflict of interest

The authors declare no conflict of interest.

## Author contributions

BM performed most of the experiments, data curation, formal analysis, methodology, conceptualization, writing original draft, review and editing. PB performed the CAM assay. TM took part in performing the mice wound‐healing assay. SC supervised and performed the mice wound‐healing assay. MD conceptualized the project, designed experiments, planned and guided the research, formal analysis, supervision, funding acquisition, resources, writing‐review and editing the manuscript.

## Supporting information


**Fig. S1.** Reduced FRG1 levels enhance tumorigenic properties in HUVECs.
**Fig. S2.** Ectopic levels of FRG1 reduce tubulogenic properties in HUVECs.
**Fig. S3.** Inhibition of FGFR reduce tumorigenic properties in HUVECs.

**Table S1.** List of primary antibodies used in Western blots.

**Table S2.** List of qRT‐PCR primers.

## Data Availability

All the data generated and analyzed in the study are included in the article and its supplementary files.

## References

[1] Sung H , Ferlay J , Siegel RL , Laversanne M , Soerjomataram I , Jemal A and Bray F (2021) Global cancer statistics 2020: GLOBOCAN estimates of incidence and mortality worldwide for 36 cancers in 185 countries. CA A Cancer J Clin 71, 209–249. 10.3322/caac.21660 33538338

[2] Lewis CE , Leek R , Harris A and McGee JO (1995) Cytokine regulation of angiogenesis in breast cancer: the role of tumor‐associated macrophages. J Leukoc Biol 57, 747–751. 7539028 10.1002/jlb.57.5.747

[3] Badodekar N , Sharma A , Patil V , Telang G , Sharma R , Patil S , Vyas N and Somasundaram I (2021) Angiogenesis induction in breast cancer: a paracrine paradigm. Cell Biochem Funct 39, 860–873. 34505714 10.1002/cbf.3663

[4] Giantonio BJ , Catalano PJ , Meropol NJ , O'Dwyer PJ , Mitchell EP , Alberts SR , Schwartz MA and Benson AB (2007) Bevacizumab in combination with oxaliplatin, fluorouracil, and leucovorin (FOLFOX4) for previously treated metastatic colorectal cancer: results from the eastern cooperative oncology group study E3200. J Clin Oncol 25, 1539–1544. 17442997 10.1200/JCO.2006.09.6305

[5] Haibe Y , Kreidieh M , El Hajj H , Khalifeh I , Mukherji D , Temraz S and Shamseddine A (2020) Resistance mechanisms to anti‐angiogenic therapies in cancer. Front Oncol 10, 221. 32175278 10.3389/fonc.2020.00221 PMC7056882

[6] Yadav V , Zhang X , Liu J , Estrem S , Li S , Gong X‐Q , Buchanan S , Henry JR , Starling JJ and Peng S‐B (2012) Reactivation of mitogen‐activated protein kinase (MAPK) pathway by FGF receptor 3 (FGFR3)/Ras mediates resistance to vemurafenib in human B‐RAF V600E mutant melanoma. J Biol Chem 287, 28087–28098. 22730329 10.1074/jbc.M112.377218 PMC3431627

[7] Manchado E , Weissmueller S , Morris JP , Chen C‐C , Wullenkord R , Lujambio A , de Stanchina E , Poirier JT , Gainor JF , Corcoran RB *et al*. (2016) A combinatorial strategy for treating KRAS‐mutant lung cancer. Nature 534, 647–651. 27338794 10.1038/nature18600 PMC4939262

[8] Lieu C , Heymach J , Overman M , Tran H and Kopetz S (2011) Beyond VEGF: inhibition of the fibroblast growth factor pathway and Antiangiogenesis. Clin Cancer Res 17, 6130–6139. 21953501 10.1158/1078-0432.CCR-11-0659 PMC5562355

[9] Casanovas O , Hicklin DJ , Bergers G and Hanahan D (2005) Drug resistance by evasion of antiangiogenic targeting of VEGF signaling in late‐stage pancreatic islet tumors. Cancer Cell 8, 299–309. 16226705 10.1016/j.ccr.2005.09.005

[10] Hanel ML , Wuebbles RD and Jones PL (2009) Muscular dystrophy candidate gene FRG1 is critical for muscle development. Dev Dyn 238, 1502–1512. 19097195 10.1002/dvdy.21830 PMC2964887

[11] Tiwari A , Pattnaik N , Mohanty Jaiswal A and Dixit M (2017) Increased FSHD region gene1 expression reduces *in vitro* cell migration, invasion, and angiogenesis, *ex vivo* supported by reduced expression in tumors. Biosci Rep 37, BSR20171062. 28947680 10.1042/BSR20171062 PMC5665614

[12] Tiwari A , Mukherjee B , Hassan MK , Pattanaik N , Jaiswal AM and Dixit M (2019) Reduced FRG1 expression promotes prostate cancer progression and affects prostate cancer cell migration and invasion. BMC Cancer 19, 346. 30975102 10.1186/s12885-019-5509-4 PMC6458714

[13] Mukherjee B , Tiwari A , Palo A , Pattnaik N , Samantara S and Dixit M (2022) Reduced expression of FRG1 facilitates breast cancer progression via GM‐CSF/MEK‐ERK axis by abating FRG1 mediated transcriptional repression of GM‐CSF. Cell Death Discov 8, 442. 36329016 10.1038/s41420-022-01240-w PMC9633810

[14] Wuebbles RD , Hanel ML and Jones PL (2009) FSHD region gene 1 (*FRG1*) is crucial for angiogenesis linking FRG1 to facioscapulohumeral muscular dystrophy‐associated vasculopathy. Dis Model Mech 2, 267–274. 19383939 10.1242/dmm.002261 PMC2675802

[15] Kumar D , Patel SA , Khan R , Chawla S , Mohapatra N and Dixit M (2022) IQ motif‐containing GTPase‐activating protein 2 inhibits breast cancer angiogenesis by suppressing VEGFR2–AKT signaling. Mol Cancer Res 20, 77–91. 34615693 10.1158/1541-7786.MCR-20-1044

[16] Naik M , Brahma P and Dixit M (2018) A cost‐effective and efficient Chick ex‐Ovo CAM assay protocol to assess angiogenesis. Methods Protoc 1, 19. 31164562 10.3390/mps1020019 PMC6526448

[17] Laddha AP and Kulkarni YA (2019) VEGF and FGF‐2: promising targets for the treatment of respiratory disorders. Respir Med 156, 33–46. 31421589 10.1016/j.rmed.2019.08.003

[18] Chen P‐Y , Simons M and Friesel R (2009) FRS2 via fibroblast growth factor receptor 1 is required for platelet‐derived growth factor receptor β‐mediated regulation of vascular smooth muscle marker gene expression. J Biol Chem 284, 15980–15992. 19339244 10.1074/jbc.M809399200 PMC2708892

[19] Cai T‐Y , Zhu W , Chen X‐S , Zhou S‐Y , Jia L‐S and Sun Y‐Q (2013) Fibroblast growth factor 2 induces mesenchymal stem cells to differentiate into tenocytes through the MAPK pathway. Mol Med Rep 8, 1323–1328. 24008926 10.3892/mmr.2013.1668

[20] Lau M‐T , So W‐K and Leung PCK (2013) Fibroblast growth factor 2 induces E‐cadherin Down‐regulation via PI3K/Akt/mTOR and MAPK/ERK signaling in ovarian cancer cells. PLoS ONE 8, e59083. 23554977 10.1371/journal.pone.0059083 PMC3598697

[21] Folkman J (1995) Angiogenesis in cancer, vascular, rheumatoid and other disease. Nat Med 1, 27–30. 7584949 10.1038/nm0195-27

[22] Holmgren L , O'Reilly MS and Folkman J (1995) Dormancy of micrometastases: balanced proliferation and apoptosis in the presence of angiogenesis suppression. Nat Med 1, 149–153. 7585012 10.1038/nm0295-149

[23] Parangi S , O'Reilly M , Christofori G , Holmgren L , Grosfeld J , Folkman J and Hanahan D (1996) Antiangiogenic therapy of transgenic mice impairs de novo tumor growth. Proc Natl Acad Sci USA 93, 2002–2007. 8700875 10.1073/pnas.93.5.2002 PMC39899

[24] Esquivel‐Velázquez M , Ostoa‐Saloma P , Palacios‐Arreola MI , Nava‐Castro KE , Castro JI and Morales‐Montor J (2015) The role of cytokines in breast cancer development and progression. J Interferon Cytokine Res 35, 1–16. 25068787 10.1089/jir.2014.0026 PMC4291218

[25] Zheng Q , Li X , Cheng X , Cui T , Zhuo Y , Ma W , Zhao X , Zhao P , Liu X and Feng W (2017) Granulocyte‐macrophage colony‐stimulating factor increases tumor growth and angiogenesis directly by promoting endothelial cell function and indirectly by enhancing the mobilization and recruitment of proangiogenic granulocytes. Tumour Biol 39, 101042831769223. 10.1177/1010428317692232 28240048

[26] Bergers G and Hanahan D (2008) Modes of resistance to anti‐angiogenic therapy. Nat Rev Cancer 8, 592–603. 18650835 10.1038/nrc2442 PMC2874834

[27] Szymczyk J , Sluzalska KD , Materla I , Opalinski L , Otlewski J and Zakrzewska M (2021) FGF/FGFR‐dependent molecular mechanisms underlying anti‐cancer drug resistance. Cancer 13, 5796. 10.3390/cancers13225796 PMC8616288 34830951

[28] Winter SF , Acevedo VD , Gangula RD , Freeman KW , Spencer DM and Greenberg NM (2007) Conditional activation of FGFR1 in the prostate epithelium induces angiogenesis with concomitant differential regulation of Ang‐1 and Ang‐2. Oncogene 26, 4897–4907. 17297442 10.1038/sj.onc.1210288

[29] Hui Q , Jin Z , Li X , Liu C and Wang X (2018) FGF family: from drug development to clinical application. Int J Mol Sci 19, 1875. 29949887 10.3390/ijms19071875 PMC6073187

[30] Zhou Y , Wu C , Lu G , Hu Z , Chen Q and Du X (2020) FGF/FGFR signaling pathway involved resistance in various cancer types. J Cancer 11, 2000–2007. 32127928 10.7150/jca.40531 PMC7052940

[31] Fitzsimons RB , Gurwin EB and Bird AC (1987) Retinal vascular abnormalities In facioscapulohumeral muscular dystrophy: a general association with genetic and therapeutic implications. Brain 110, 631–648. 3580827 10.1093/brain/110.3.631

[32] Akl MR , Nagpal P , Ayoub NM , Tai B , Prabhu SA , Capac CM , Gliksman M , Goy A and Suh KS (2016) Molecular and clinical significance of fibroblast growth factor 2 (FGF2 /bFGF) in malignancies of solid and hematological cancers for personalized therapies. Oncotarget 7, 44735–44762. 27007053 10.18632/oncotarget.8203 PMC5190132

[33] Jia T , Jacquet T , Dalonneau F , Coudert P , Vaganay E , Exbrayat‐Héritier C , Vollaire J , Josserand V , Ruggiero F , Coll J‐L *et al*. (2021) FGF‐2 promotes angiogenesis through a SRSF1/SRSF3/SRPK1‐dependent axis that controls VEGFR1 splicing in endothelial cells. BMC Biol 19, 173. 34433435 10.1186/s12915-021-01103-3 PMC8390225

[34] Pepper MS , Ferrara N , Orci L and Montesano R (1992) Potent synergism between vascular endothelial growth factor and basic fibroblast growth factor in the induction of angiogenesis in vitro. Biochem Biophys Res Commun 189, 824–831. 1281999 10.1016/0006-291x(92)92277-5

[35] Shin M , Beane T , Quillien A , Male I , Zhu LJ and Lawson ND (2016) Vegfa signals through ERK to promote angiogenesis, but not artery differentiation. Development 143, 3796–3805. 27578780 10.1242/dev.137919 PMC5087643

[36] Gerber H‐P , McMurtrey A , Kowalski J , Yan M , Keyt BA , Dixit V and Ferrara N (1998) Vascular endothelial growth factor regulates endothelial cell survival through the phosphatidylinositol 3′‐kinase/Akt signal transduction pathway. J Biol Chem 273, 30336–30343. 9804796 10.1074/jbc.273.46.30336

[37] Morales‐Ruiz M , Fulton D , Sowa G , Languino LR , Fujio Y , Walsh K and Sessa WC (2000) Vascular endothelial growth factor–stimulated Actin reorganization and migration of endothelial cells is regulated via the serine/threonine kinase Akt. Circ Res 86, 892–896. 10785512 10.1161/01.res.86.8.892

[38] Ibrahimi OA , Zhang F , Lang Hrstka SC , Mohammadi M and Linhardt RJ (2004) Kinetic model for FGF, FGFR, and proteoglycan signal transduction complex assembly. Biochemistry 43, 4724–4730. 15096041 10.1021/bi0352320

[39] Ansari MJ , Bokov D , Markov A , Jalil AT , Shalaby MN , Suksatan W , Chupradit S , AL‐Ghamdi HS , Shomali N , Zamani A *et al*. (2022) Cancer combination therapies by angiogenesis inhibitors; a comprehensive review. Cell Commun Signal 20, 49. 35392964 10.1186/s12964-022-00838-y PMC8991477

