# Supplementary material for: Reduced FRG1 expression promotes angiogenesis via activation of the FGF2‐mediated ERK/AKT pathway
Source: FEBS Open Bio. 2023 Mar 31;13(5):804–17. doi: 10.1002/2211-5463.13582 (PMC10153342; doi:10.1002/2211-5463.13582)
Supplement: Supplementary file 1 — Fig. S1. Reduced FRG1 levels enhance tumorigenic properties in HUVECs. Fig. S2. Ectopic levels of FRG1 reduce tubulogenic properties in HUVECs. Fig. S3. Inhibition of FGFR reduce tumorigenic properties in HUVECs. [file FEB4-13-804-s002.pdf]

Supplementary Fig. S1: Reduced FRG1 levels enhance tumorigenic properties in HUVECs

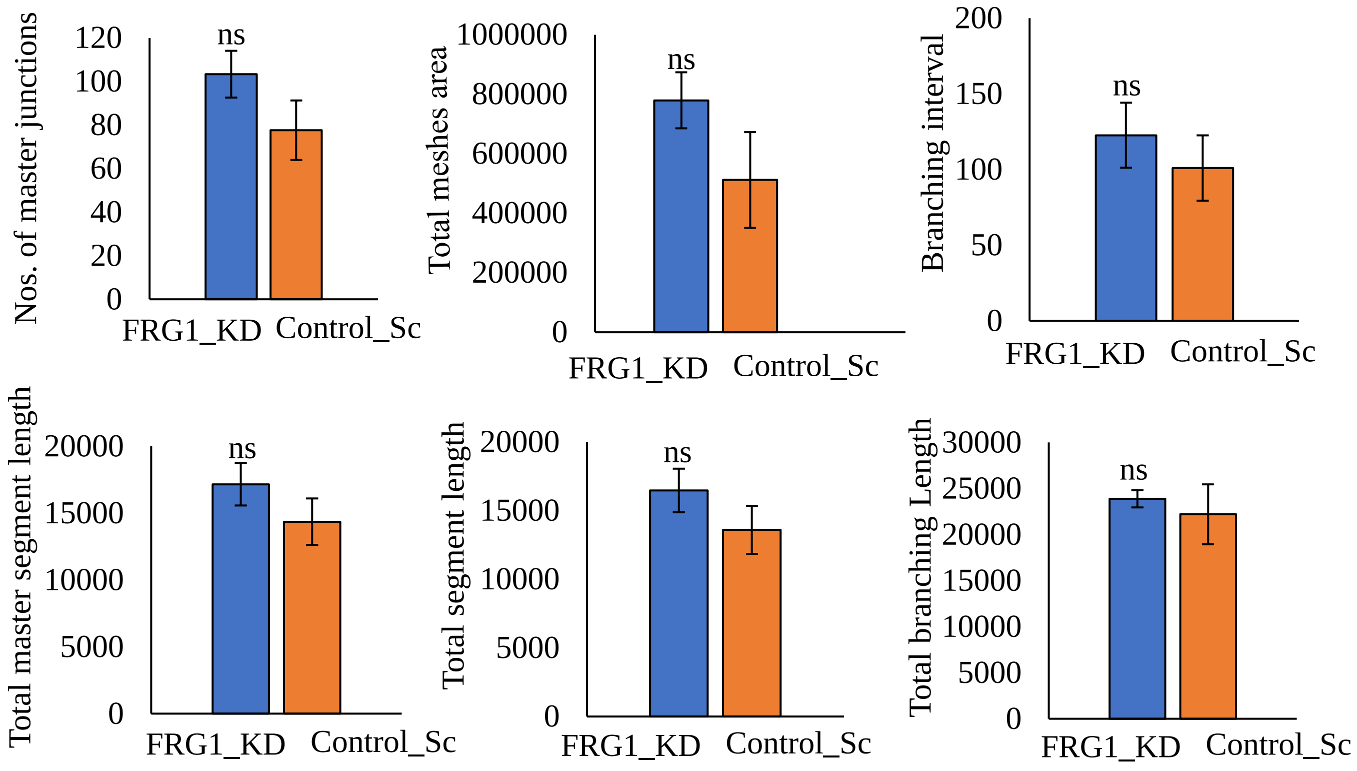

Supplementary Fig. S1. Decrease FRG1 level increases the tubulogenic ability of HUVECs. HUVECs were grown in the conditioned media (CM), harvested from MCF7 cells with depleted FRG1 (FRG1_KD) and Control_Sc. After 6 hours of incubation, images of the tubules were captured at X4 magnification (given in Figure 2A) and further analyzed by imageJ software. Bar diagram showing the difference in number of master junctions, total master segment length, total meshes area, total branching length, total segment length, branching interval in MCF7_KD group vs. Control_Sc. Experiments were performed in triplicate. Two-tailed unpaired student's t-test was used to compare the differences between groups. Results are presented as mean ± SD. ns, $p > 0.05$.

Supplementary Fig. S2: Ectopic levels of FRG1 reduce tubulogenic properties in HUVECs

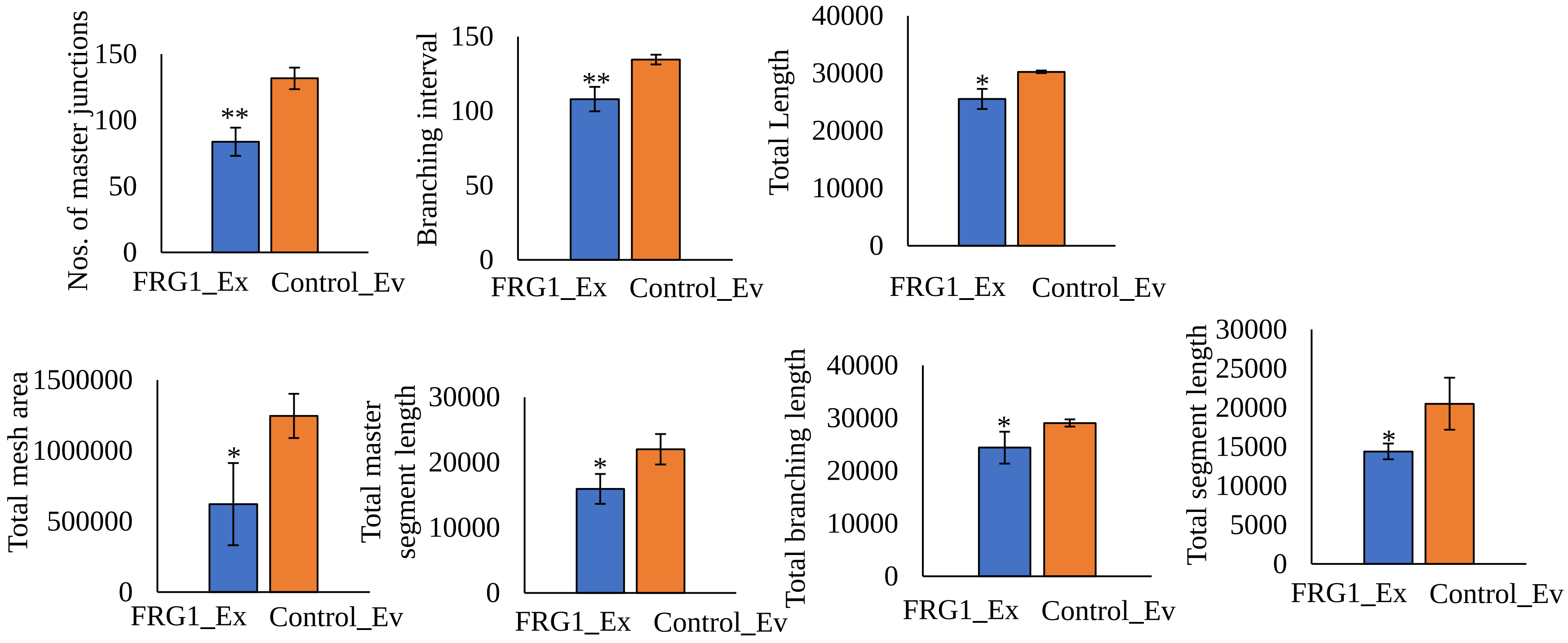

Supplementary Fig. S2. Elevated level of FRG1 level decreases the tubulogenic ability of HUVECs. HUVECs were grown in the conditioned media (CM), harvested from MDAMB-231 cells with ectopic expression of FRG1 (FRG1_Ex) and Control_Ev. After 6 hours of incubation, images of the tubules were captured at X4 magnification (given in Figure 2C) and further analyzed by imageJ software. Bar diagram showing the difference in number of number of master junctions, total master segment length, total meshes area, total branching length, total length, total segment length, branching interval, in MCF7_Ex group vs. Control_Ev. Experiments were performed in triplicate. Two-tailed unpaired student's t-test was used to compare the differences between groups. Results are presented as mean ± SD. *, $p \leq 0.05$; **, $p \leq 0.01$.

Supplementary Fig. S3: Inhibition of FGFR reduces tumorigenic properties in HUVECs

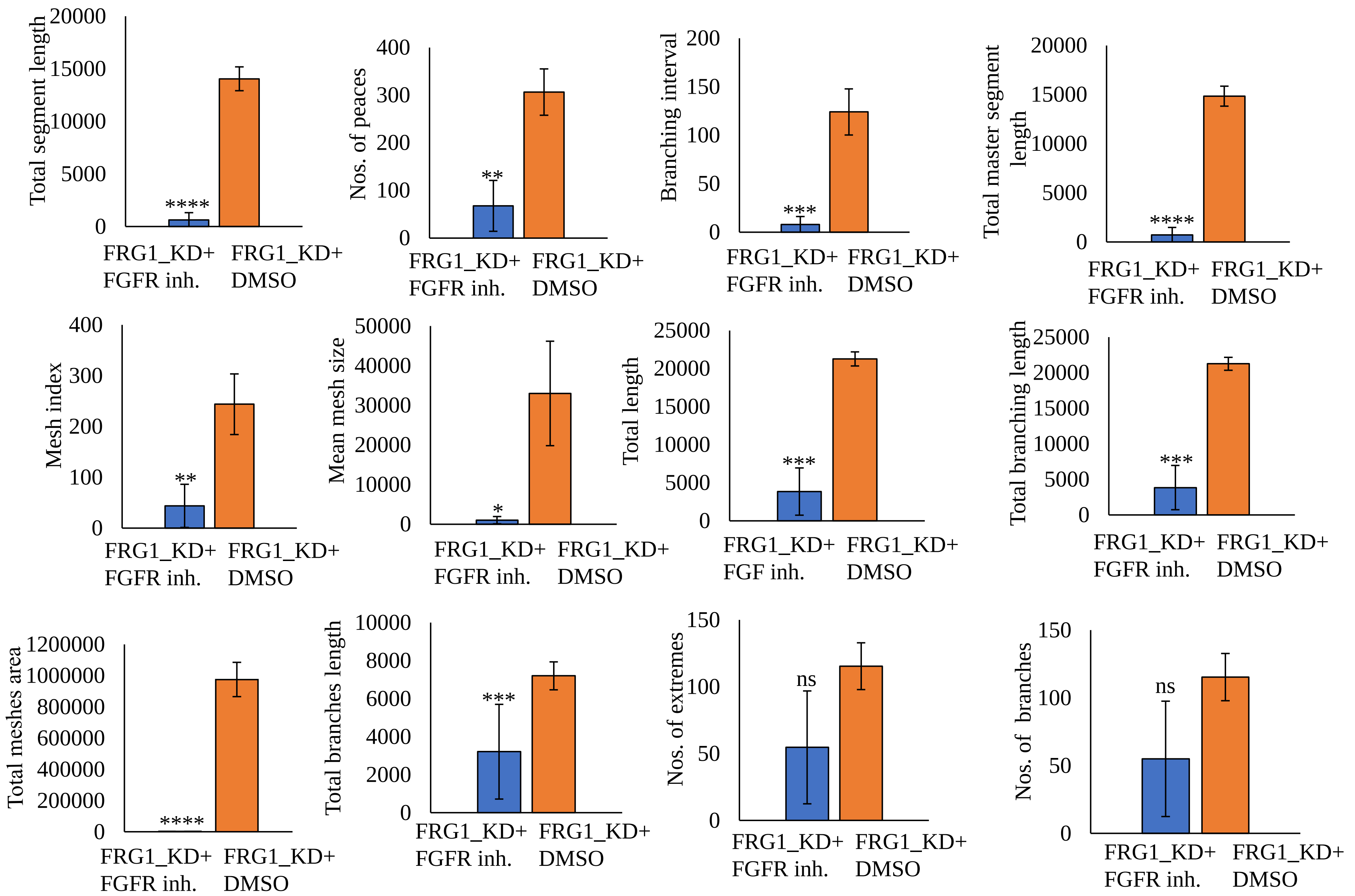

Supplementary Fig. S3. FGFR inhibition in FRG1 depleted MCF7 cells reduces tubule formation ability of HUVECs. Conditioned media (CM) was collected from MCF7 cells with depleted FRG1 expression (CM_FRG1_KD) and the corresponding control (Control_Sc), and used for growing HUVECs along with of FGF receptor (FGFR) inhibitor Infigratinib (100 nm) or DMSO control for 4 hours. After 6 hours of incubation, images of the tubules were captured at X4 magnification (given in Figure 6B) and further analyzed by imageJ software. Bar graphs showing various tubulogenic parameters in the two groups. Experiments were performed in triplicate, two-tailed unpaired student's t-test was used to compare the two groups' differences. Results are presented as mean ± SD. ns, $p > 0.05$, *, $p \leq 0.05$; **, $p \leq 0.01$; ***, $p \leq 0.001$, ****, $p \leq 0.0001$.
