# Supplementary material for: Reduced FRG1 expression promotes angiogenesis via activation of the FGF2‐mediated ERK/AKT pathway
Source: FEBS Open Bio. 2023 Mar 31;13(5):804–17. doi: 10.1002/2211-5463.13582 (PMC10153342; doi:10.1002/2211-5463.13582)
Supplement: Supplementary file 2 — Table S1. List of primary antibodies used in Western blots. [file FEB4-13-804-s003.docx]

**Supplementary Table 1:** List of primary antibodies used in Western blots

| **S. No.** | **Name of antibody** | **Dilution** | **Brand** |
| --- | --- | --- | --- |
| 1 | Anti- FRG1 | 1:10000 | Abcam, MA, USA |
| 2 | Anti-phospho-ERK | 1:1000 | CST, MA, USA |
| 3 | Anti-phospho-AKT 308 | 1:1000 | CST, MA, USA |
| 4 | Anti-phospho-AKT 473 | 1:1000 | CST, MA, USA |
| 5 | Anti-GAPDH | 1:10000 | Abgenex, India |
