# Supplementary material for: Reduced FRG1 expression promotes angiogenesis via activation of the FGF2‐mediated ERK/AKT pathway
Source: FEBS Open Bio. 2023 Mar 31;13(5):804–17. doi: 10.1002/2211-5463.13582 (PMC10153342; doi:10.1002/2211-5463.13582)
Supplement: Supplementary file 3 — Table S2. List of qRT‐PCR primers. [file FEB4-13-804-s001.docx]

**Supplementary Table 2:** List of qRT-PCR primers

| **S. No.** | **Primer Name** | **Primer sequences (5' - 3')** |
| --- | --- | --- |
| 1 | VEGF A F | ATCTGCATGGTGATGTTGGA |
| 2 | VEGF R | GGGCAGAATCATCACGAAGT |
| 3 | GAPDH F | ACCCAGAAGACTGTGGATGG |
| 4 | GAPDH R | TCTAGACGGCAGGTCAGGTC |
| 5 | FGF2 F | GCTGTACTGCAAAAACGGGG |
| 6 | FGF2 R | TAGCTTGATGTGAGGGTCGC |
